# Regulation and criminal law enforcement in healthcare: an uneasy alliance or partners in crime?

**DOI:** 10.1186/s12913-026-14381-w

**Published:** 2026-03-18

**Authors:** Quirine Amelink, Jan-Willem Weenink, Ian Leistikow

**Affiliations:** 1 Erasmus School of Health Policy & Management, Erasmus University Rotterdam, Burgemeester Oudlaan 50, Rotterdam, 3062PA The Netherlands; 2 Department of Legal Affairs, Health & Youth Care Inspectorate, Postbus 2115, Utrecht, 3500GC The Netherlands; 3 Department of Executive support, Policy & Strategy, Health & Youth Care Inspectorate, Postbus 2115, Utrecht, 3500GC The Netherlands

**Keywords:** Healthcare, Regulation, Criminal law enforcement, Collaboration, Interdisciplinarity, Patient safety

## Abstract

This paper reports on research on the collaboration between the regulator and criminal law enforcement in the healthcare sector. Based on observations of consultations between the regulator and criminal law enforcement in The Netherlands, two underlying factors are distinguished that create challenges in this collaboration: practical issues and ideological differences. The divergent ideologies and values between regulation and criminal law enforcement hinder an integrated approach to incidents in healthcare. We argue that articulating and valuing these ideological differences would create an opportunity for mutual disciplinary enrichment. An interdisciplinary approach to incidents in healthcare could mitigate the inherent biases of both actors and thus provide otherwise unseen opportunities to prevent similar future incidents. By doing so, the regulator and criminal law enforcement could work towards a form of collaboration regarding incidents in healthcare that does justice to the interests of both the patients, the healthcare sector and society.

## Background

Regulatory authorities and criminal law enforcement regularly perform their duties in the same area. This is quite common in, for example, the taxation sector and environmental protection. In the healthcare sector, criminal law enforcement and the regulatory authority are also regularly confronted with cases that touch on both domains. There are numerous examples of behavior in healthcare that could lead to an intervention by the regulator as well as to criminal prosecution, such as sexual abuse in a healthcare setting, the illegal trade in pharmaceutical products, and healthcare administration fraud.

In addition, infliction of harm by healthcare workers might mobilize both the regulator and criminal law enforcement, as it is often unclear whether the harm caused is deliberate, an adverse event in care delivery or a medical complication. Since it is not unusual that patients pass away while in treatment, mortality or injury due to deliberate harm could go unnoticed for quite some time. Examples of this are cases like those of Lucy Letby and Charles Cullen, both nurses found guilty of murdering multiple patients during their professional practice. These cases were extensively covered in the media and caused a shock in society [1, 2]. At the same time, in most of the cases where patients are harmed or die, this was not the intention of the healthcare professional even if it was the result of their actions. Such situations could also lead to the criminal prosecution of healthcare professionals, as shown in the cases of RaDonda Vaught and Hadiza Bawa-Garba. It is up for debate whether it is justified to prosecute a healthcare professional after adverse events, including a lethal error [3, 4]. The foregoing cases raise the question how adverse events that lead to the death or injury of a patient should be approached by the authorities involved. On the one hand, people receiving healthcare are vulnerable to healthcare professionals with malicious intentions. This stresses the importance of taking the possibility that patients could be deliberately harmed by healthcare professionals into account. On the other hand, the fact that healthcare professionals’ acts will unintentionally lead to harm or even death of a patient could be seen as an occupational hazard of healthcare delivery, and more often is rather the cause of system flaws than individual wrongdoing. In light of the above, opinions may differ on what could be seen as a justified response and which authority should be intervening in such cases, the regulator or the prosecutor. These considerations are also relevant for other types of incidents in healthcare, such as sexual misconduct and fraud. Sexual misconduct in a healthcare setting is not rare [5]. While in some cases it is clear that the vulnerable position of a patient is being abused by a healthcare professional, there may also be cases where this is less obvious. Certain forms of physical examination may be perceived as a sexual violation even though a sexual intention is lacking. Healthcare fraud has also been receiving a lot of attention in recent years [6]. It may be unclear at first glance whether someone is deliberately committing fraud for personal gain, bending the rules in the interest of the patient, or is incompetent to comply with the often complex administrative financial regulations in healthcare.

The foregoing indicates the importance of coordination between regulatory authorities and criminal law enforcement in the healthcare sector. Effective coordination and collaboration between the regulatory authorities and criminal law enforcement can contribute to responses after incidents in healthcare that do justice to those involved, whether this is criminal prosecution or a regulatory intervention. However, research on this specific type of collaboration is currently lacking in literature. At the same time, previous literature does suggest that there are opportunities for mutual disciplinary enrichment when it comes to regulatory and criminological insights [7].

The coordination and collaboration between the regulatory authority and criminal law enforcement in the healthcare sector has already received some attention in The Netherlands. The Netherlands Public Prosecution Service (*Openbaar Ministerie*) and the Dutch Health & Youth Care Inspectorate (*Inspectie Gezondheidszorg en Jeugd*) regularly work on the same cases. Their cooperation is based on a so-called ‘Healthcare Cooperation Protocol’ (*Samenwerkingsprotocol Gezondheidszorg*). This protocol was comprehensively revised after a tragic case in 2013 regarding a general practitioner. After violating end of life care protocols and legislation he was charged with murder by the Public Prosecution Service (PPS) and at the same time the Health & Youth Care Inspectorate (HYI) imposed a public order to stop the general practitioner from working. The general practitioner committed suicide during the investigations. The case caused great concern among healthcare professionals. The Dutch Parliament concluded that the communication between the PPS and the HYI had to improve [8]. Another case that raised questions about the coordination between PPS and the HYI was the so-called ‘insulin murders’. In 2017, a Dutch healthcare worker was arrested and charged with the murder of multiple nursing home residents by the unnecessary administration of insulin. He was found guilty of killing four people. The healthcare worker had been able to harm residents in several nursing homes between September 2016 and November 2017 [9]. In December 2017, a nursing home found a connection between the unnecessary administration of insulin and the healthcare worker. They reported this to the police and the PPS started a criminal investigation. Earlier, the HYI had received incident reports of two nursing homes about patients with inexplicable low blood sugars. One of the reports stated that there were suspicions about an intern, which later turned out to be the arrested healthcare worker. The HYI had not asked additional questions about this suspicion, but had assessed that there had probably been a medication error and closed the investigation [10].

The abovementioned cases highlight the importance of coordination and collaboration between regulatory authorities and criminal law enforcement in the healthcare sector, because they are illustrative for the dilemmas involved in assessing incidents in healthcare and the choice for either a regulatory or criminal law approach. They illustrate that, whatever the choice may be, the outcome of that choice can have a huge impact on those involved, the healthcare sector and society. This paper reports on research into the coordination and collaboration between the regulatory body and criminal law enforcement in Dutch healthcare. The aim of this study was to explore how this collaboration works out in practice. A second objective was to explore what possible consequences the way they collaborate could have for the pursuit of an integrated approach to incidents in healthcare.

## Methods

### Setting

The Health and Youth Care Inspectorate (HYI) is the regulatory body in The Netherlands and constitutes the state supervision of the quality of both healthcare professionals and providers. The HYI is authorized to inspect healthcare providers, announced or unannounced, and investigate the quality of care. Also, healthcare providers in The Netherlands are legally obliged to report certain incidents to the HYI: adverse events that lead to the death or serious injury of a patient, (sexual) violence in the care relationship and the dismissal of a healthcare professional due to poor performance. In 2023 the HYI received around 5.000 (health)care related reports, and around 11.000 reports related to pharmaceutical products or medical devices [11]. In addition, it is also possible for civilians and other organizations to report alarming situations in healthcare to the HYI. In most cases, the healthcare provider involved is ordered by the HYI to investigate the incident. The healthcare provider must send an investigation report to the HYI describing the incident, possible causes, and improvement measures taken to prevent similar incidents in the future. The HYI then assesses the quality of the investigation and the suitability of the proposed improvement measures. If the investigation report meets the quality requirements, the procedure will be closed. When the HYI suspects that there is a possible threat to the safety of patients, the HYI will also investigate the incident itself [12, 13]. If the HYI deems it necessary, it can take administrative measures to ensure compliance to laws and regulations by the healthcare provider. The HYI is also legally entitled to lodge complaints about healthcare professionals to a disciplinary board. In The Netherlands, the (professional) conduct of registered healthcare professionals can be reviewed by a disciplinary board (*Regionaal en Centraal Tuchtcollege voor de Gezondheidzorg*) which has the power to impose measures that may restrict the professional practice of the healthcare professional.

**Fig. 1:**
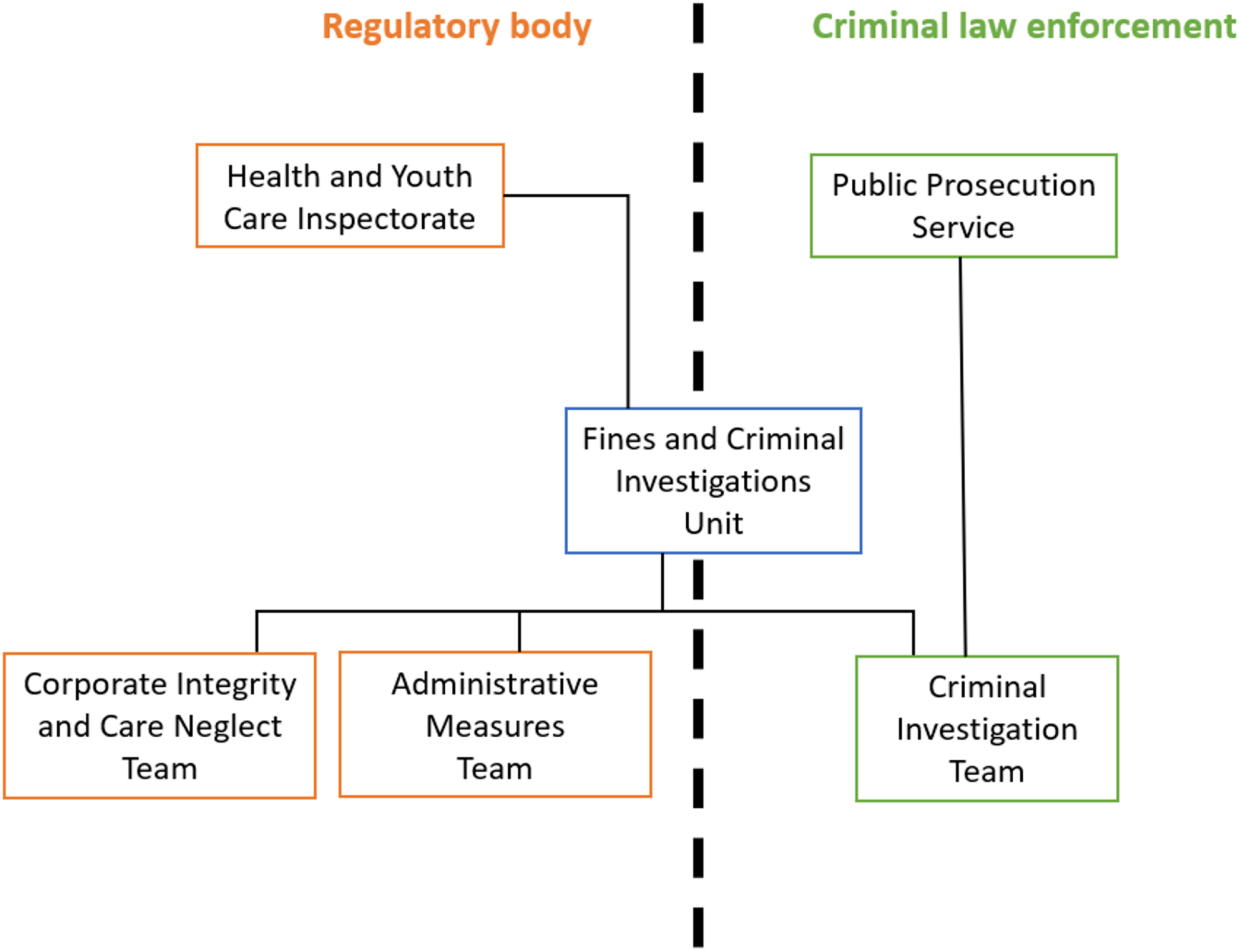
Organization chart HYI and PPS regarding criminal investigations

During the abovementioned procedures the HYI is regularly confronted with behavior of healthcare professionals that could be qualified as a criminal offence. The HYI houses a Fines and Criminal Investigations Unit (*Bureau Opsporing en Boetes*). The Criminal Investigation Team of this unit investigates possible criminal offences in healthcare under the direction of the Dutch Public Prosecution Service. While this unit is part of the HYI, the members of the Criminal Investigation Team are so-called special investigating officers (*buitengewoon opsporingsambtenaren*) and operate on different authorities than the inspectors of the HYI do. Their authorities are based on criminal law and police law, while the authorities of inspectors are based on administrative law or disciplinary law (Fig. 1).

The Netherlands Public Prosecution Service (PPS) is responsible for investigating and prosecuting criminal offences on behalf of society. The main tasks of the PPS are: supervising the police in the investigation of criminal offences, prosecuting criminal offences, out-of-court settlement or bringing suspected offenders before the courts. The PPS houses a Department of Medical Affairs (*Expertisecentrum Medische Zaken*) that provides advice and guidance in criminal investigations or prosecution in the healthcare sector. In addition, at each public prosecution office in The Netherlands a so-called medical public prosecutor is appointed. The medical public prosecutor is primarily in charge of all healthcare related cases of that particular office.

The Healthcare Cooperation Protocol serves as the foundation for the collaboration between the HYI and the PPS. The protocol describes two forms of consultation between the two organizations. First of all, there is an annual meeting at management level to discuss (joint) policy and priorities. Second, on a case level, consultation takes place during a so-called Three Party Consult (*Tripartite Overleg*). During these meetings the HYI, the Criminal Investigations Team of the HYI and the PPS discuss cases that they are investigating and where mutual coordination might be required. During a Three Party Consult (TPC) the organizations could exchange expertise, such as medical expertise or expertise regarding a criminal investigation. A TPC could also be used to coordinate the nature and timing of the actions that are deployed by the HYI or the PPS, in order to prevent obstruction of one the investigations and to strive for the most effective, efficient and proportionate deployment of regulatory and law enforcement instruments and interventions. Finally, in a TPC the organizations could explore which substantial information is helpful for each other’s investigation and whether it could be exchanged on a legal basis [14]. During a TPC, procedural or more general information can be shared. However, in order to be able to use substantial information coming from the other party in an investigation, the parties must formally exchange that information in line with legal requirements. The TPC’s are organized by the Criminal Investigations Team of the HYI at the request of the HYI or the PPS when deemed necessary by either organization.

It is important to point out that in the Netherlands the exchange of medical data and (confidential) information about patients is strictly regulated. In The Netherlands, healthcare professionals must adhere to a confidentiality obligation with regard to the information they have obtained in the course of their professional practice. It means that, without the permission of the patient, they are not allowed to share information regarding a patient with anyone, other than healthcare professionals that are directly involved in providing care for that patient. In addition to this, healthcare professionals have a so-called legal privilege, meaning that they cannot be obliged to make statements as a witness and to provide information in criminal procedures. Persons working in administrative or supporting positions within a healthcare provider are subject to a so-called derived confidentiality obligation and derived legal privilege. They are not allowed to decide whether to provide medical data to third parties, not even in criminal procedures. This decision is reserved for the primary confidentiality holder. Officials of the HYI are, by law, subject to the same derived confidentiality obligation and derived legal privilege.

### Study design, data collection and analysis

We employed a research design using an ethnographic approach to study how the collaboration between the HYI and PPS works out in practice. Observation is at the core of ethnographic research and was therefore chosen as the method to collect data [15]. This approach allows to obtain insight into the internal processes of a group and organization, and it helps to recover the distinct meaning given to these processes by the actors involved [16].

Over the course of thirteen months (April 2022 – May 2023), the first author conducted observations of eighteen TPCs to study the collaboration between the HYI and PPS. We included all TPCs that were organized during the study period and were logistically feasible for the first author to attend, with the goal of retrieving the broadest possible data. In total, 18 out of 27 TPCs that were organized during our study period were included.

The observational method used was on-site nonparticipant observation [15]. The first author did not interfere in the meeting by asking questions or seek clarification to minimize the impact on the observed setting and maintain objectivity. Disrupting a TPC by having the first author ask questions was not appropriate due to the urgency and sensitivity of the cases being discussed. The first author went into the observations with an open and broad approach and paid attention to multiple elements of the TPCs, such as the atmosphere, substantive dialogues and discussions, the underlying procedures and the dynamics between the persons participating in the meeting. During the observations it was made clear to all participants that the first author was present as a researcher and was making field notes. Written informed consent has not been obtained since the process of signing would have disturbed the situation that was to be observed. Also, it was not always clear in advance who would be present during a to be observed TPC. To respect the confidential nature of the meetings observed, no audio or video recordings were made. Instead, the field notes were drawn up into observation reports after the observations to safeguard as many details as possible. In the fieldnotes, the names of the people, healthcare providers and other organizations involved were not included to protect their privacy. Therefore, no personal data was collected. In addition, verbal informed consent was always obtained from all participants present.

The analysis focused on exploring overarching and recurring themes in the data [17]. The fieldnotes of the observations were analyzed while data collection continued. Fieldnotes were inductively coded by the first author using Atlas.ti in order to identify common patterns in the data. These patterns included observed elements and mechanisms related to the collaboration between the HYI and PPS, such as the topics of the discussions that took place during the TPCs as well as the attitude of the attendees and the arguments they put forward. The first author conducted a thematic analysis by following these steps: data familiarization, generating initial codes, searching for themes, defining and naming themes and subthemes [18]. Themes and subthemes were formed by the first author by grouping the codes, which led to the creation of a coding scheme. This coding scheme was used during the further analysis of the fieldnotes. During this process, the themes and subthemes were discussed, reviewed and revised by the first, second and third author during multiple meetings, which led to adapting and improving the coding scheme. The data was analyzed, with the purpose of this study in mind, in order to understand and reflect on the different elements that were distinguished regarding the collaboration between the PPS and HYI. We focused on the goals of the collaboration between the PPS and HYI, but we also included the nature of the arguments put forward by the attendees during the discussions, the atmosphere of the meetings and the dynamics between the attendees. In the analysis, we also paid attention to whether the subject of the case being discussed affected the aforementioned aspects. Our analysis led to three main themes related to the collaboration between the HYI and PPS in the healthcare sector.

### Reflexivity

It is appropriate to point out that during the fieldwork study the first author was employed as a legal adviser at the HYI. Therefore, the first author had easy access to data sources and had helpful insider-knowledge of processes and procedures. Without this existing relationship it would not have been possible to observe the TPCs. At the same time, this insider-knowledge and the very close proximity of the first author to the studied setting and the people involved, increased the risk of being biased. To minimize this risk, regular meetings to critically reflect on the findings were held with the other two authors who were not involved in the legal department of the HYI. Furthermore, the observer saw that the way participants in TPCs interacted was not influenced by her presence, as this was identical to how they interacted in previous TPCs the first author attended in her role as legal adviser. Finally, it is important to point out that during the observed TPCs the first author did not provide legal advice and was therefore present only in the role of researcher.

## Results

Eighteen TPCs that took place between April 2022 and May 2023 were observed during this study. Thirteen TPCs were held in a digital meeting. Four TPCs took place at the office of the HYI and one took place at an office of the PPS. The duration of the TPCs varied from 30 min up to 1,5 h. Each TPC was chaired by an official of the HYI Criminal Investigation Team and an administrative official of this team kept notes of the meeting. Furthermore, the attendees of the observed TPCs were one or two inspectors and a legal advisor of the HYI as well as an official of the Department of Medical affairs and a Public Prosecutor of the PPS. At some TPCs, involved police detectives, secretaries of the PPS and managers and a policy advisor of the HYI were also present. During the TPCs, cases were discussed that both the HYI and PPS were investigating or had been investigating. Nine cases were about professional misconduct by a healthcare professional, five cases about sexual misconduct or violence committed by a healthcare professional, one case about fraud in the healthcare sector and three cases about violations of medical product legislation. In the following paragraphs, we will discuss our findings on how the collaboration between the PPS and HYI worked in practice. We have categorized our findings according to the three main themes that we found regarding the collaboration between the HYI and PPS: exchanging information and expertise, coordinating investigative actions and interventions, and underlying values.

### Exchanging information and expertise

In this paragraph we describe what the organizations exchange during TPCs. First, we found that the PPS had a substantially greater need for expertise from the HYI than vice versa. Second, we found that the PPS often faced a lack of information during their investigations. The HYI usually did have all the information necessary for their investigation. The HYI was often unable to provide the PPS with substantive case information due to legal restrictions regarding confidentiality of medical data, while the PPS was frequently willing and able, by law, to share this type of information with the HYI.

Expertise was exchanged regularly during a TPC. Inspectors of the HYI usually had healthcare-related knowledge, especially about norms regarding professional standards and quality indicators. At the same time, prosecutors and other officials of the PPS had more expertise in the area of criminal law and criminal proceedings. Prosecutors and officials of the Department of Medical Affairs of the PPS often appealed to the healthcare-related knowledge of the inspectors, mostly when it concerned an investigation about professional misconduct or medical product legislation.

*The prosecutor explains that the PPS waited for the investigations by the HYI and the Ministry regarding this particular medical device*,* before making a decision about possible prosecution. He says that the PPS would like to make that decision now*,* but in order to do so it wants to have a full picture of the situation. The prosecutor refers to a memo in which several questions for the HYI were written down. The chairman asks if the HYI would like to respond to this. The inspector takes the floor and indicates that she has prepared a presentation because she would like to provide some context regarding medical devices and the applicable legislation.* (Observation no. 8).

The inspectors seemed very willing to share their expertise on healthcare-related subjects with the PPS. While providing explanation about healthcare related subjects, they frequently used medical or healthcare jargon that was unknown to PPS officials. As a result, officials of the PPS regularly asked for clarification.

*The inspector explains possible complications that may occur and states that certain complications are a common phenomenon. The inspector uses a lot of medical jargon in the explanation. The inspector repeats the questions from the Public Prosecution Service*,* which were already submitted prior to the consultation*,* and provides quick and brief answers. The chairman asks the Public Prosecutor if she is satisfied with the answers. The Public Prosecutor responds and says that she is*,* but that the explanation went very quickly. She asks some clarifying questions. *(Observation no. 17)

Inspectors seem to have less need for the expertise from the PPS. The questions of the inspectors were mainly focused on the status of the criminal investigation or prosecution.

The PPS regularly announced that they lacked information to successfully investigate the case concerned. In particular, this lack of information involved medical data of the patient involved. The HYI however, was often very well informed and commonly had access to relevant medical data.

*The legal advisor of the HYI says that her perception is that this patient knew what he wanted and had a clear plan about which he only informed the doctor. The employee of the PPS says they have very limited medical records and do not have enough information to establish that. The HYI legal advisor asks a number of other questions. The employee of the PPS reiterates that they only have a very limited file and therefore she cannot answer the questions asked.* (Observation no. 10).

While the PPS regularly indicated during TPCs that they lacked relevant information, it seemed that the derived confidentiality obligation regarding medical information hindered the inspectors from providing useful information that they held to the PPS.

*The legal advisor of the HYI explains that they cannot answer this question either. She explains that the HYI often has to deal with medical data and that medical data is subject to a derived confidentiality obligation. The HYI legal advisor says that the fact that somebody is a patient at a certain institution is already considered to be medical data*,* which makes sharing information difficult.* (Observation no. 3).

*The inspector says the situation at this healthcare provider has improved and mentions that the inspection reports will be made public*,* referring to the timeframe by which she expects the reports to be on the website. The inspector asks if this raises any questions. The Public Prosecutor responds and laughingly says that such an explanation always raises questions. The prosecutor says he understands the inspector’s position but mentions that she spoke in a lot of generalities and asks if a little more explanation is possible.* (Observation no. 9).

Inspectors primarily shared process-level information and not substantive case information. As mentioned above, the inspectors did often share their expertise on healthcare-related subjects. However, this was done by speaking in generalities and not going into the specifics of the case.

*The chairman asks if there are any questions. The inspector says he is curious whether this TPC has yielded anything for the PPS. The Public Prosecutor says she has become wiser. According to her*,* context is important in this kind of case. She says that for instance*,* it is good to know how things are arranged in the national healthcare field and that this is important for the PPS’s consideration of the follow-up. She mentions that this does not even require sharing specific information about the case.* (Observation no. 14).

On the other hand, the PPS often seemed very willing to share specific and substantive case information with the HYI if it could help their investigation.

*The inspector points out that the HYI can currently only focus its investigation on what has been reported and not on the new information of the PPS. According to the inspector*,* this information can only be included if the PPS formally reports it to the HYI. The prosecutor indicates that she is willing to formally report it.* (Observation no. 6).

Provision of information by the HYI to the PPS in cases of professional misconduct and (sexual) violence committed by a healthcare professional against a client seemed to be quite difficult as a result of the derived confidentiality obligation regarding medical data. At the same time, in these types of cases, the PPS often indicated to have a lack of information. The foregoing seemed to be much less of an issue in cases involving fraud and violations of medical product legislation, since no client information or medical data had to be shared.

### Coordinating investigative actions and interventions

In this paragraph, we describe how the PPS and HYI coordinate their investigative actions and interventions during a TPC. Regarding the joint consideration of an appropriate intervention, the PPS regularly inquired about and encouraged an intervention by the HYI, which often was not the HYI’s intended action. Finally, we found that during TPCs the organizations expressed different views on what is a just response towards the subject of the investigation.

During TPCs, the PPS and HYI regularly discussed their (future) investigative actions and considered whether their investigations would not be obstructed by each other’s actions and therefore agreements had to be made.

*The inspector points out that the investigation of the HYI could take quite long and that in her opinion the PPS is better able to ensure the safety of healthcare and patients in the meantime.* (Observation no. 1).

The PPS asked the HYI from time to time to exercise some restraint in its investigation by, for example, not talking to the suspect. Sometimes the PPS indicated that talking to the suspect by the HYI was possible but required that the HYI did not mention the ongoing criminal investigation. In general, the HYI was prepared to go along with this. If the HYI considered that there was too great a risk to patient safety, it indicated to the PPS that it would continue its investigation. The PPS did not refute that the HYI had to be able to intervene in such cases.

*One of the detectives responds and indicates that as a detective she prefers to have as few other investigations as possible in addition to the criminal investigation. She indicates that the suspect knows that something is going on and that an investigation by the HYI will not come as a surprise. At the same time*,* the suspect does not yet know that a criminal investigation is being conducted. The legal advisor of the HYI asks if she can conclude from this that they are allowed to talk to the suspect*,* but that they are not allowed to mention that the HYI knows that a criminal investigation is being conducted. The detective confirms this and says: “You are concerned about this case and so are we. It would be strange if you could not do your work because we are stopping you from doing so."* (Observation no. 3).

We found that during some TPCs, the HYI and PPS explored which interventions could be deployed for the organizations involved. For example, it was discussed which intervention would be more appropriate and which organization should intervene.

*The chairman asks if the HYI can also intervene after this violation* [diploma forgery]. *The legal advisor of the HYI explains that an administrative fine could possibly be imposed. However*,* according to her*,* this depends on what actions the PPS will take and whether that might conflict with each other.* (Observation no. 7).

In some TPCs, the HYI expressed hope that the PPS would prosecute a case, since they found criminal prosecution appropriate.

*The inspector continues and explains that this investigation led to the report of a criminal offence by the HYI*,* hoping that something would be done with it. According to the inspector*,* imposing an administrative fine has little effect in this type of case. They think and hope that criminal law can send a strong signal though.* (Observation no. 13).

We found that in cases of professional misconduct the PPS regularly called for action from the HYI. In some cases, the PPS indicated that an intervention by the HYI may ensure that criminal prosecution could be avoided.

*The Public Prosecutor expresses his incomprehension as to why a disciplinary procedure is not initiated by the HYI and he says that now they have no choice but to prosecute. According to the Public Prosecutor*,* criminal prosecution is more serious for a healthcare professional than disciplinary law and therefore the Public Prosecution Service would like to be able to refrain from criminal prosecution.* (Observation no. 4).

*The Public Prosecutor says that he has a strong suspicion of assisted suicide*,* but he also takes into account the circumstances of the case and the position of the physician. The Public Prosecutor explains that he has the possibility to refrain from prosecution and to stop the criminal investigation. With this TPC*,* he would like to explore how the HYI assesses these types of cases. The chairman adds the question whether criminal law is appropriate in these types of cases or perhaps another route.* (Observation no. 10).

The PPS sometimes seemed to struggle with the question whether criminal prosecution is appropriate after investigating a case of professional misconduct, while they did feel that some kind of intervention was necessary. The PPS regularly stated that disciplinary law or administrative law was the preferred route in these cases, as this would constitute a legal reason for them to refrain from criminal prosecution and to close the case.

*The Public Prosecutor says that she is particularly interested in the actions that the HYI will take. This could help her with a possible decision to dismiss the case and explain this to the relatives of the deceased patient.* (Observation no. 12).

At the same time, our findings suggest that the HYI often did not seem willing to initiate disciplinary proceedings or administrative enforcement in cases of professional misconduct. By the time the TPC took place, in many cases the HYI already had a clear idea about which intervention they would or would not deploy.

*The manager of the PPS asks whether filing a complaint to the disciplinary board is being considered by the HYI. The inspector responds by saying that in this case it was a joint action of the involved professionals and therefore she is not inclined to do so. She also states that there is a limited risk to patient safety because the violation* [violating regulations for medical scientific research with people] *has already stopped. * (Observation no. 2).

In some cases of professional misconduct, inspectors of the HYI were surprised that the PPS was even considering criminal charges at all and sometimes empathized with the involved healthcare professionals or healthcare provider. On the other hand, in a case of violation of medical product legislation the inspectors and legal advisor of the HYI were unpleasantly surprised that the PPS did not want to initiate criminal proceedings, and instead encouraged the HYI to take action, as the HYI felt that clearly a criminal offence had been committed.

*The inspector takes the floor and says he keeps coming back to the licensing requirement not being met. The Public Prosecutor says that this is indeed the case*,* but that he doubts whether a criminal prosecution will have any effect. He does not consider this case suitable for criminal prosecution and believes it should be addressed through healthcare quality law. In this*,* he seems to be steering very clearly towards a role for the HYI. The legal advisor of the HYI responds and says she does not understand the PPS’s reluctance. She doubts that it is up for discussion whether the conduct discussed is a criminal offence. It seems completely clear to her that it is.* (Observation no. 11).

There seemed to be lack of trust in each other’s methods and assessments. In a number of TPCs, inspectors of the HYI called into question whether criminal prosecution was appropriate in the discussed case about professional misconduct.

*The manager of the PPS says that they have to assess the case because it may concern multiple deaths. The legal advisor of the HYI counters that it has not been established at all that the deaths are related to the violation* [violating regulations for medical scientific research with people]. *The manager of the PPS starts to contradict this. The inspector interrupts him and says that multiple factors played a role in the deaths of the patients.* (Observation no. 2).

On the other hand, in quite a few TPCs, officials of the PPS were questioning the methods of the HYI for investigating a case. In response to this, inspectors of the HYI sometimes seemed to be taking on a defensive attitude when explaining why they have or have not taken certain actions.

*The HYI legal advisor then says that in this case they did not speak to the healthcare professional themselves. She seems a little reluctant to admit this. The Public Prosecutor asks why the healthcare professional was not spoken to. The inspector explains that when an adverse event is reported*,* the board of the healthcare provider is the point of contact*,* and that it is unusual to speak to the individual healthcare professionals. It seems like the inspector is trying to defend himself. The Public Prosecutor says that he wonders when the HYI will make the transition from focusing on the improvement of future healthcare towards the fact that someone actually died*,* and mistakes were made. (Observation no. 4). *

The foregoing findings illustrate that the HYI and PPS seemed to have different views on when criminal prosecution in the healthcare sector is appropriate. We found that during TPCs about professional misconduct and violations of medical product legislation this led to a lot of discussion with opposing views. The PPS and HYI also seemed to have different views on when it is suitable to initiate administrative enforcement or file a complaint to the disciplinary board in cases of professional misconduct and violations of medical product legislation. During TPCs about cases of (sexual) violence inflicted by a healthcare professional or fraud, this kind of discussion hardly ever arose.

### Underlying values

The PPS and HYI seemed to have different attitudes towards the healthcare sector. In addition, the PPS and HYI seemed to have a different view on allocation of responsibility for incidents in healthcare.

Inspectors of the HYI often expressed their trust in the good intentions of people working within healthcare. They expect and believe that healthcare providers are intrinsically motivated to implement improvement measures after incidents.

*The inspector of the department of mental health takes the floor and says that the healthcare provider carried out investigations that were of good quality and that the reports contained clear conclusions and adequate improvement measures. He says that they have therefore chosen to close the case. The inspector of the department of specialized medical care says he endorses this. * (Observation no. 18).

In several TPCs, the inspectors appeared to defend the actions of a healthcare provider or healthcare professional if the PPS took a critical position, especially in cases of professional misconduct.

*According to the HYI manager*,* the hospital’s misjudgment led to this violation and they themselves also admit that they made an error of judgement. Immediately*,* she links this to the full intensive care units during the COVID-19 period when this violation took place. The inspectors very quickly indicate that they agree with the manager. The HYI manager emphasizes that the system worked well in this case*,* because the hospital found the error itself and reported it immediately*,* submitting an investigation report to the HYI. She also emphasizes that the hospital has been very open and transparent and has done a good job of learning and improving. She reiterates that the hospital itself acknowledged the assessment error and underlines that the hospital has seen the offence as common practice*,* in which COVID-19 also played a major role. *(Observation no. 2).

The PPS seemed to have a different attitude. They expressed more distrust of the healthcare sector and were more critical and sometimes somewhat suspicious towards the healthcare providers and professionals involved in the cases discussed.

*The official of the Department of Medical affairs of the PPS responds and says she really cannot follow the healthcare provider’s conclusion that this was no culpable adverse event. She describes the patient’s trauma and that it clearly fits with a fall. The Public Prosecutor takes the floor and again explains that they are concerned that the healthcare provider has written a very nice investigation report*,* but that they will not follow-up on it. The inspector shakes her head. *(Observation no. 15).

The PPS sometimes seemed frustrated when healthcare providers did not voluntarily cooperate in providing them with medical data and invoked the right of non-disclosure arising from their confidentiality obligation. This behavior was considered by the PPS as willingly frustrating the criminal investigation.

*The Public Prosecutor starts talking about the healthcare provider frustrating the criminal investigation. In doing so*,* the prosecutor again mentions the medical data that the healthcare provider initially refused to provide. The prosecutor feels that the healthcare provider itself should have considered that there were very exceptional circumstances and should have cooperated*,* given that they indicated they were so shocked by the case. The prosecutor seems to be distrustful of the healthcare provider here. The prosecutor explains what the next steps will be in the criminal investigation and what obstacles she expects to encounter. Again*,* she refers to the fact that the healthcare provider reported the incident quite late. The prosecutor says that the PPS could have done more if they had been notified earlier. She is highly critical of the healthcare provider’s conduct. *(Observation no. 5).

The HYI and PPS seemed to have different views on the allocation of responsibility and accountability when investigating incidents in healthcare. When investigating an incident, particularly a clinical adverse event, the inspectors of the HYI were primarily looking at the system in which the incident occurred. They seemed to pay little attention to possible individual culpability.

*The inspector says that this is related to the question whether criminal prosecution is appropriate in these types of cases. He indicates that providing care involves more than just following guidelines and protocols and that sometimes incorrect assessments are made and sometimes the course of the disease is unpredictable. This is all inherent to an adverse event*,* according to the inspector. The official of the Department of Medical Affairs of the PPS indicates that it must always be a case-by-case assessment and that various facts and circumstances can play a role. She gives the example that further investigation* [into an individual] *could be carried out if several things have gone wrong. The inspector responds and says that several things going wrong is precisely what fits the description of an adverse event because if one of the safety valves in the system had worked*,* the adverse event would not have occurred. *(Observation no. 12).

The PPS reasoned much more from the concept of individual accountability rather than the functioning of a system, also when it came to cases about professional misconduct in a healthcare setting.

*The Public Prosecutor asks why the role of the individual is not looked at by the HYI when investigating clinical adverse events. He says that a clinical adverse event involves human error*,* and therefore it would be very logical to look at the individual. The inspector repeats a theoretical explanation about clinical adverse events in which failing of the system plays a major role. The Public Prosecutor asks why the system as well as the individual cannot be looked at during an investigation and indicates that the HYI also has a task in disciplinary law. *(Observation no. 4).

Investigation by the HYI seemed to be almost exclusively focused on ensuring patient safety and the improvement of quality of care in the future. The PPS, and the use of criminal law in general, seemed to have the additional goal to achieve a form of retribution for the intentional or culpable crimes committed.

*The inspector says she understands that relatives of deceased patients may feel that the HYI is not doing enough*,* as they do not always deploy additional measures after investigating serious medical error*,* but that satisfaction for injured parties does not play a role in the task of the HYI as a regulatory body. *(Observation no. 16).

The different attitudes of the PPS and HYI towards the healthcare sector and healthcare professionals and their opposing views regarding the allocation of accountability were especially visible during TPCs about professional misconduct and less during TPCs about (sexual) violence by a healthcare professional, fraud and violations of medical product legislation.

## Discussion

Our empirical study reveals that challenges in the collaboration between the regulatory body and criminal law enforcement in healthcare influence the possibility to pursue an integrated approach in assessing incidents. We first provide a brief methodological reflection before we reflect on our findings.

### Strengths and limitations

A strength of this study is the uniqueness of the data. The TPCs observed are confidential and not accessible to the public. The first author was employed as a legal adviser at the HYI during the study and therefore had access to data sources and insider-knowledge of processes and procedures. This provided a unique opportunity to collect this specific data. The study also has some limitations. First, the data that was collected by observing eighteen TPCs that all took place within about one year, which could be seen as a snapshot. However, during this time frame all the TPCs where the first author could be present were observed. This retrieved a broad dataset for that period. Furthermore, we noticed data saturation after the first fifteen TPCs observed. We started the analysis of the fieldnotes while the data collection was still taking place. During the thematic analysis conducted by the first author, meetings were held with the second and third author to discuss, review and revise the defined themes. After discussing the analysis of fieldnotes on fifteen TPCs, we noticed that the same themes kept recurring. This presumed data saturation was confirmed by the last three TPCs observed and analyzed, since no new themes and subthemes emerged. Additionally, during our analysis we found themes that appear in international literature on public sector collaboration and are not particularly time-specific. Therefore, we believe that the data collected represents a sufficiently accurate picture of the cooperation between the regulator and criminal law enforcement in healthcare in The Netherlands. Second, when using the on-site nonparticipation observational method, no clarifying or in-depth questions are asked. This possibly complicates a deeper understanding of the observed situation and the meanings or thoughts that direct behavior. However, due to the sensitivity of the cases discussed, the TPCs could only be observed if the TPCs would not be disturbed by the observation. Furthermore, the first author, because of her position as a legal adviser at the HYI, was acquainted with the setting and had considerable knowledge about the dynamics, practices and agreements that played a role in the situations observed. Therefore, the observations led to a profound understanding of the cooperation between the HYI and the PPS, without needing to ask clarifying questions. Future research could offer deeper insights into the intentions, thoughts and values that influence behavior in the collaboration between the HYI and PPS. Finally, generalizing these findings across and within similar settings outside of The Netherlands needs to be done with caution. The characteristics of collaboration between regulatory bodies and criminal law enforcement depend on the national context and legislation. Although these will differ internationally, we do believe our findings are of international relevance as the themes we identified relate to previous international findings about public-sector collaboration and the dilemmas involved in choosing criminal prosecution or regulatory interventions after incidents in healthcare [3, 4, 19]. How these mechanisms play out in each country might differ and could be input for future (comparative) research.

### Unequal access to information and expertise

Our findings show an asymmetry in both expertise and information available to the regulator and criminal law enforcement, which made an integrated approach of cases challenging. The HYI had more expertise regarding clinical diagnosis and treatment which it openly shared with the PPS. The PPS was often willing to share substantive information about their criminal investigation with the HYI. The HYI had substantial (medical) information at its disposal that it could not share with the PPS due to legal restrictions, even when the PPS needed that information. The PPS often had difficulties obtaining medical information for criminal investigations due to regulations on (derived) confidentiality. As a result, the PPS and HYI often spoke in generalities during TPCs. This asymmetry regarding the possession of medical information seems to challenge their collaboration. Mayer and Kenter have argued that having shared resources is a critical element to the overall success of collaboration in achieving organizational goals. According to them, the pooling of resources is one of the primary reasons parties agree to collaborate since shared resources lead to the creation of something greater than any individual could produce on their own [19]. Being able to share expertise and to have access to the same information could be seen as a form of shared resources. We found that the regulator and criminal law enforcement often are not able to use the same information and that this frustrates their collaboration. We also saw, as previously described by O’Leary & Vij, power imbalances due to the unequal access to resources [20]. This seems to be particularly frustrating in cases concerning professional misconduct, in which medical information plays an important role.

### Different attitudes and underlying values

Our findings show that, during the TPCs, the regulator and criminal law enforcement inform each other about how they assess the case discussed based on their own perspective and possibilities, but that they do not come to a discussion leading to a mutual approach for the most effective and appropriate intervention. The PPS often indicated that if the HYI would initiate an administrative or disciplinary intervention, they could refrain from pressing criminal charges. At the same time, the HYI was often reluctant to deploy such regulatory interventions because they did not consider these supportive for improving the quality and safety of future care. This led to frustration on the side of the PPS because although they were not eager to press criminal charges, they did feel that an intervention was called for. The regulator and criminal law enforcement seemed to act and argue on the basis of different underlying values with regard to responding to incidents in healthcare. This was especially visible in cases about professional misconduct, and particularly related to three aspects: (1) a focus on trust versus distrust, (2) a focus on individuals versus the system, and (3) a focus on retribution versus improvement.

First, the HYI often expressed their trust in the good intentions of healthcare professionals, whereas the PPS seemed have a somewhat distrustful attitude. The nature of their work makes both actors prone to bias. Where criminal law enforcement primarily sees individuals who behave unlawfully to achieve some form of personal gain, regulators primarily see individuals who unintentionally become involved in incidents due to flaws of the system they work in. When confronted with serious harm or death of a patient, criminal law enforcement might be biased towards individual culpability while the regulator might be biased towards system failure. Research has shown that trust in the relationship between regulators and regulatees has a positive effect on regulatee compliance and therefore helps safeguard public interests and control public risks [21]. Trust is considered important since an opportunity to correct mistakes occurs when there is trust on both sides—when regulators believe the regulatee is competent and good-willed and the regulatee in turn is willing to acknowledge errors because they expect to be treated fairly [22]. Nevertheless, it is also important that the regulator ensures the protection of clients from healthcare providers who do not act with good intentions, with whom a relationship of trust does not contribute to the effectiveness of the regulator.

Second, we found that an investigation by the HYI primarily focused on how the system should be organized to prevent similar incidents in the future and that a criminal investigation by the PPS focused on the allocation of culpability for a criminal offence by, most of the time, one or more individuals. Almond and Van Erp have described the different perspectives arising from the theories behind regulation and criminal law enforcement and argue that both disciplines could draw insights from one another. For example, regulation could adopt insights from criminal law enforcement into agency and individual motivations, broadening the scope from the institutionalist, or systems-oriented, approach predominant in regulation. At the same time, the systems-oriented approach could provide new insights for criminal law enforcement in addition to their perspectives on individual accountability [7]. Literature on regulation promotes the systems-oriented approach and individual corrective actions are considered weaker than solutions targeting the system level [23]. The theory behind this is that a lack of compliance by individuals usually reflects impractical policies in the context of poorly designed systems. In-depth investigations after adverse events should identify underlying cognitive, task, environmental, workflow, organizational or other system factors that contributed to policy noncompliance [24]. It is even argued that the pursuing of individual culpability can only be justified when healthcare professionals demonstrate serious professional misconduct, sexual abuse of patients, use of mind-altering substances, commit criminal offences or intentionally harm patients [25]. Although this seems hard to argue against, the difficulty lies in assessing whether a specific situation indeed fits the criteria of individual culpability or system failure. In light of the foregoing, it is arguable that the regulator should keep the possibility of individual culpability in mind, and that the addition of a systems-oriented approach to criminal law investigations could potentially prevent criminal prosecution of well-intentioned healthcare professionals who made a mistake [3, 4].

Finally, it seems that the goals the regulator and criminal law enforcement pursue differ. The regulator strives for healthcare providers to be able to learn and improve to best ensure patient safety in the future. Criminal law enforcement aims to protect patients from criminal behavior by identifying and punishing offenders [26, 27]. In safety literature, concepts like just culture and restorative justice have been proposed as means to further enhance quality and safety in healthcare. Reasons to support such an approach include the idea that it offers a more holistic approach to healing, and that most incidents in healthcare involve no deliberate wrongdoing and are interwoven with systemic failures [28, 29]. Avoiding retribution after incidents in healthcare may also prevent health professionals’ deviation from sound medical practice in fear of malpractice litigation or for other self-protective motives (defensive medicine) known to have a negative impact on the quality of care [30, 31]. Moreover, research has shown that the use of sanctions of any form as part of an incident analysis process damages the ability of an organization to learn and improve [32]. Therefore, some regulators are moving away from retribution as instrument and are incorporating the concepts of just culture and restorative justice into their practice [33]. Criminal law enforcement however, considers retribution as an essential instrument to protect society from crime. Offender accountability and retribution are fundamental goals of criminal law [26, 27]. Although both regulators and criminal law enforcement aim to improve the quality and safety of future care and patient safety, their beliefs about how to best achieve this conflict.

### A shared approach for criminal law enforcement and regulation

Being able to have access to the same resources is, as already described in this discussion, seen as an important element to the overall success of collaboration [19]. Our findings show that the limitations arising from legislation on access to medical information create an information and power asymmetry between the collaborating parties. Establishing an integrated approach to incidents in healthcare is thus complicated by the fact that regulators and criminal law enforcement often do not have access to the same (amount of) information.

Having a shared vision and common interest also plays an important role in making collaboration successful [19]. According to Gajda and Koliba, common interest is often the first link that brings stakeholders together and it creates a sense of group ownership of a certain issue [34]. Ferreyra and Beard describe that at that point the common interest should be transformed to a shared vision and strategy and goals, in which stakeholder values, perspectives and expectations are included [35]. A broadly shared vision should motivate stakeholders to contribute resources and work together to overcome difficulties [34, 36]. Gray and Margerum describe that ideological obstacles could inhibit consensus building in collaboration [37, 38]. Furthermore, according to Mayer and Kenter, trust is seen as one of the most vital components necessary to build and sustain collaboration [19]. McNamara has described that trust between partners in interorganizational collaborations is based on mutual understanding and the confidence that the stakeholders are working toward collective action [39]. According to Lasker et al., a lack of trust significantly decreases the likelihood of a collaborative being successful [40]. The contradicting underlying values that we found, potentially make it hard for the collaborating organizations to agree on whether an intervention by the regulator or criminal law enforcement should prevail. After all, if there is no consensus on which response is appropriate and a certain degree of trust in each other’s methods and procedures, there is little chance that either organization will feel comfortable in letting the other decide on the appropriate intervention. Different ideologies can, as literature suggests and our findings show, hinder shared decision-making and an integrated approach. Criminal law enforcement and regulation in healthcare could potentially benefit from each other’s different approaches and insights, but our findings suggest that in practice it complicates their collaboration. This seems like a missed opportunity, because appreciating the other’s perspective could help mitigate the inherent biases of both actors.

## Conclusions

The regulator and criminal law enforcement are faced with challenges in their collaboration in the healthcare sector. We have identified a number of elements that may underlie these challenges. These broadly fall into two categories: practical and ideological. Practical issues, like unequal access to medical information due to legislation, complicate the possibility of the regulator and criminal law enforcement to make a joint decision after incidents in healthcare based on a shared level of information. Ideological differences, for example on the value of individual accountability or retribution, also hinder the regulator and criminal law enforcement to come to an integrated approach to incidents in healthcare. Particularly when it concerns incidents regarding professional misconduct. Despite these differences, our observations showed a common drive to improve healthcare quality and patient safety as well as a commitment of both parties to collaborate. Consideration should be given at the policy and legislative levels to how the regulator and criminal law enforcement should work together in the healthcare sector and what shared resources, such as information, and (legal) arrangements are needed to achieve successful cooperation. Furthermore, instead of only discussing the cases, the regulator and criminal law enforcement could make each other’s ideologies and motivations more explicit and value them as a way to mitigate biases and obtain relevant new insights. This could increase mutual understanding and develop these ideological differences from being an obstacle to becoming a strength. By doing so, the regulator and criminal law enforcement can work towards a form of collaboration regarding incidents in healthcare that does justice to the interests of both the patients, the healthcare sector and society.

## Data Availability

The datasets generated and/or analyzed during the current study are not publicly available due to sensitivity of the data but are available from the corresponding author on reasonable request.

## Abbreviations

PPS: Public Prosecution Service
HYI: Health & Youth Care Inspectorate
TPC: Three Party Consult
